# Axenic *in vitro* cultivation and genome diploidization of the moss *Vesicularia montagnei* for horticulture utilization

**DOI:** 10.3389/fpls.2023.1137214

**Published:** 2023-03-20

**Authors:** Yong Hu, Qing Li, Zexi Chen, Zhanwu Xu, Hongyu Li, Congfa Wen, Liu Duan, Hong Yang, Li Liu

**Affiliations:** ^1^ State Key Laboratory of Biocatalysis and Enzyme Engineering, Hubei Collaborative Innovation Center for Green Transformation of Bio-Resources, Hubei Key Laboratory of Industrial Biotechnology, School of Life Sciences, Hubei University, Wuhan, Hubei, China; ^2^ Department of Economic Plants and Biotechnology, Yunnan Key Laboratory for Wild Plant Resources, Kunming Institute of Botany, Chinese Academy of Sciences, Kunming, China; ^3^ University of Chinese Academy of Sciences, Beijing, China; ^4^ Lishui Runsheng Moss Technology Co., Ltd. Green Valley Information Industrial Park, Lishui, Zhejiang, China

**Keywords:** diploidization, moss, cultivation, horticulture, Vesicularia montagnei

## Abstract

Mosses are widely used in the establishment of greenery. However, little research has been conducted to choose a suitable species or improve their performance for this application. In our study, we examined *Vesicularia montagnei* (*V. montagnei*), a robust moss that is widely distributed in temperate, subtropical, and tropical Asia with varying environmental conditions. Axenic cultivation system of *V. montagnei* was developed on modified BCD medium, which enabled its propagation and multiplication *in vitro*. In this axenic cultivation environment, several diploid *V. montagnei* lines with enhancement of rhizoid system were generated through artificial induction of diploidization. Transcriptomic analysis revealed that several genes responsible for jasmonic acid (JA) biosynthesis and signaling showed significant higher expression levels in the diploid lines compared to the wild type. These results are consistent with the increasement of JA content in the diploid lines. Our establishment of the axenic cultivation method may provide useful information for further study of other *Vesicularia* species. The diploid *V. montagnei* lines with improved rhizoid system may hold promising potential for greenery applications. Additionally, our study sheds light on the biosynthesis and functions of JA in the early landed plants.

## Introduction

Mosses are increasingly being used in the development of greenery in city buildings and indoor environments. The green landscape in cities has beneficial impact on psychological well-being because lacking of vegetation in cities may lead to lower social and mental health issues (Kuo and Sullivan, 2001). In addition to these benefits, mosses do not need fertilizer or pesticides compared with the flowering or vascular plants, making them easy to maintain (Julinova and Beckovsky, 2019). Furthermore, mosses can survive long periods of drought stress and can regenerate once they get moist again. Giving these advantages, mosses are becoming more and more popular for use in designing natural living landscapes in urban buildings and indoor environments. This includes the use of moss in vertical gardens, moss walls (Udawattha et al., 2018; Perini et al., 2022), green roofs (Anderson et al., 2010; Cruz de Carvalho et al., 2019), and bio-walls. Mosses growing on flexible materials can also be cut into customized shapes, logos, or art ornaments for indoor decoration or micro-landscape design (Julinova and Beckovsky, 2019).

Although the utilization of mosses in the greenery establishment was widely reported (Gunawardena and Steemers, 2019; Perini et al., 2022), the source of moss materials remains to be a challenge. The selected moss species collected from the wild may not be homogeneous and may contain other unknown organisms that could be harmful to humans, because moss layers are important habitats for periphyton and invertebrates (Wulf and Pearson, 2017). Furthermore, a vast amount of disruption of the wild moss layer may lead to land degradation (Adessi et al., 2021). Thus, artificial planting is the most sustainable way to obtain moss materials. Previous studies have reported on the axenic cultivation of various moss species for research purposes (Collier and Hughes, 1982; Cove et al., 2009b), commercial applications (Zhao et al., 2019; Heck et al., 2021) and rare taxa conservation (Sabovljević et al., 2012b; Sabovljević et al., 2022). However, to date, there is no report on the cultivation of *V*. *montagnei*.

These applications of mosses for establishing greenery requires a strong root-like system that can attach to the supporting surfaces. However, unlike vascular plants with a root system, mosses only possess root-like structures called rhizoids, which provide less adhesive ability. This limitation restricts their use in urban greenery establishment, particularly in the vertical applications. To overcome this issue, promising solutions include screening and selecting specific moss species suitable for this application or breeding new moss lines using biotechnological approaches.

Polyploidy refers to the presence of more than two haploid genomes in a single nucleus of an organism. This phenomenon is considered a potent evolutionary force that drives genetic variation and functional novelty (Paun et al., 2007; Hegarty and Hiscock, 2008). Polyploidization has been proven to be a useful biotechnology and is wildly used to improve agronomic traits of horticultural crops, such as *Citrus* (Aleza et al., 2009), *Lycium* (Rao et al., 2020), and *Populus* (Xu et al., 2016) plants. Phenotypic changes that occur after polyploidization include increases in leaf size, plant height, stomatal length and stress resistance (Eng and Ho, 2019). However, the induction of polyploidy in mosses for improved horticultural applications is still rare.

Jasmonic acid (JA) is a lipid-derived plant hormone that is generated from trienoic fatty acid through the octadecanoid pathway. JA is involved in regulating various aspects of plant development, including root architecture (Wasternack and Hause, 2013; Wasternack and Strnad, 2018). In Arabidopsis, the JA signaling pathway was found to inhibit root growth and the formation of lateral and adventitious roots (Wasternack and Hause, 2013). However, JA was shown to promote lateral root formation in rice (Wang et al., 2002). Compared with that in vascular plants, the effects of JA in early landed plants seem to be more complex. A study in a fern plant *Platycerium bifurcatum* revealed that low concentrations of JA (0.01-1 μM) promoted the formation and elongation of primary rhizoids, whereas concentrations exceeding 1 μM had an inhibitory effect (Camloh et al., 1996). Both cyclopentenone (+)-cis-12-oxo-phytodienoicacid (OPDA, the precursor of JA) and methyl jasmonate (JA-Me) at the concentration of 5-50 μM significantly inhibited the growth of rhizoids. However, only OPDA but not JA or its derivative JA-Ile, could be detected in the moss *P. patens* (Stumpe et al., 2010; Ponce De León et al., 2012). Nevertheless, JA has been detected in several other moss species using phytohormone profiling (Záveská Drábková et al., 2015).

## Materials and methods

### Plant materials

The original plant material of *V. montagnei* was obtained from Lishui Runsheng Moss Technology Co., Ltd. The *V. montagnei* was cultivated in greenhouse condition.

### Axenic cultivation

The sporophyte capsules of *V. montagnei* were induced under the greenhouse conditions (25°C, 16 h light, and 8 h dark). The sporophyte capsules were surface sterilized with incubation in 1 ml of 10% sodium hypochlorite solution for 7 min. The spores were released in 1 ml of autoclaved water by opening the sterilized capsules with forceps, and then transferred to Petri dish containing one of the following solid medium: (1) BCDAT medium (1 mM MgSO_4_, 10 mM KNO_3_, 45 μM FeSO_4_, 1.8 mM KH_2_PO_4_, and trace element solution (0.22 μM CuSO_4_, 0.19 μM ZnSO_4_, 10 μM H_3_BO_3_, 0.10 μM Na_2_MoO_4_, 2 μM MnCl_2_, 0.23 μM CoCl_2_, 0.17 μM KI), and 1 mM CaCl_2_, 5 mM diammonium(+)-tartrate, 0.8% (w/v) agar) according to Cove et al. (2009a), or (2) LQ #1 medium (BCDAT medium with 58 mM sucrose) or (3) LQ #2 medium (BCDAT medium with increased concentration of 20 mM KNO_3_) or (4) LQ #3 medium (BCDAT medium with 58 mM sucrose and increased concentration of 20 mM KNO_3_). Spores were germinated on the indicated media in the incubator with controlled conditions (25°C, light intensity of 60-80 μmol m^-2^ s^-1^, 16 h light, and 8 h dark).

### Polyploidization induction

Protonema (20 d after germination) of *V. montagnei* was immersed in 0.3% (w/v) colchicine solutions for 6 h on a shaker at 60 rpm/min, and then rinsed 5 times with sterile water. Treated protonema was then homogenized with a blender (ULTRA-TURRAX Tube Drive, IKA). Finally, the obtained suspension was transferred to indicated media and cultured in the incubator under the conditions described above.

### Ploidy identification

Before the ploidy identification, the treated protonema was subjected to 4 rounds of homogenization to avoid obtaining chimeric plants. Three times of ploidy analyses were conducted after the first, second and seventh subculture. First, the gametophyte moss material was cut into fragments in a pre-cooled petri dish, and 1 ml of lysate was added. After filtering into a flow tube, 25 μl of propidium iodide (1 mg/mL) fluorescence staining solution was added for 1 h in the dark, and polyploidy was subsequently detected by using a FACSCalibur Flow Cytometer (BD Biosciences, San Jose, CA, USA).

### Transcriptome analysis

The whole gametophytes of WT *V. montagnei* and diploid lines were harvested and frozen in liquid nitrogen until RNA extraction. Total RNA was extracted using TRIzol reagent (Vazyme Biotech, Nanjing, China) according to the manufacturer’s recommendations. The integrity and quality of the total RNA were verified using a 2100 Bioanalyzer RNA Nano chip (Agilent, Santa Clara, CA, USA). The concentration was measured with an ND-2000 spectrophotometer (NanoDrop, Wilmington, DE). The RNA was stored at −80°C for subsequent use.

The mRNA of each library was sequenced on an Illumina Novaseq 6000 platform located at the Major Biotech (Shanghai, China; http://www.majorbio.com/). The NT (ftp://ftp.ncbi.nlm.nih.gov/blast/db), NR (ftp://ftp.ncbi.nlm.nih.gov/blast/db), COG (http://www.ncbi.nlm.nih.gov/COG), KEGG (http://www.genome.jp/keg), and Swiss-Prot (http://ftp.ebi.ac.uk/pub/databases/swissprot) databases were used for blast search and annotation (Altschul et al., 1990). Blast2GO (v2.5.0) was used to obtain the GO (http://geneontology.org) annotation (Conesa et al., 2005), and InterProScan5 (v5.11–51.0) was used to obtain the InterPro (http://www.ebi.ac.uk/interpro) annotation (Quevillon et al., 2005). Blast similarity searches were performed for pairwise comparisons of all libraries. Orthologous and homoeologous genes were both standardized by the following criteria: E-value ≤ 9E^−100^, alignment length ≥ 200 bp, and identity ≥ 90%. Fragments per kilo base per million (FPKM) was used to estimate the expression levels of genes and to compare the differences of gene expression among samples. Differentially expressed genes (DEGs) were identified through an algorithm developed by Audic and Claverie (1997). The criterion applied was |log2Ratio| ≥ 1.0 and FDR ≤ 0.05.

### Analysis of homologs in the JA biosynthesis and signaling pathways

The proteins responsible for the JA biosynthesis pathway in Arabidopsis including Fatty Acid Desaturase (FAD), Phospholipase A1 (PLA1), Lipoxygenase (LOX), Allene Oxide Synthase (AOS), Allene Oxide Cyclase (AOC), 12-oxo-phytodienoic acid reductase (OPR3), OPC-8:0 CoA ligase (OPCL); acyl-CoA oxidase (ACX), 3-ketoacyl-CoA thiolase (KAT), Multifunctional Protein (MFP) and JASMONATERESISTANT 1 (JAR1) and proteins responsible for the JA signaling pathway in Arabidopsis including MYC2, NOVEL INTERACTOR OF JAZ (NINJA) and JASMONATE-ZIM-DOMAIN PROTEIN 1 (JAZ1) were used as queries to BLAST against the all the transcripts obtained in the analysis of the transcriptome. The results were filtered by the following criteria: E-value ≤ 9E^−10^, and identity ≥ 30%.

### Measure of the content of JA

JA were extracted from 0.1 to 0.3 g of frozen gametophytes as described by Cai et al. (2015). Stable isotope-labeled JA (D_6_-JA, 0.2 ng) was used as the internal standard for JA and added to the sample extraction buffer. Phytohormones were extracted and the content was determined by High Performance Liquid Chromatography-Tandem Mass Spectrometry (HPLC-MS/MS) using the method described by (Qi et al., 2016).

### Expression analysis with quantitative real-time PCR

Total RNA was extracted from the total gametophytes of three individual plants of WT and the diploid line using the same methods described above. Three biological replicates and three technical replicates were used for qRT-PCR analysis. Primers were designed using Beacon Designer 7.0 software (sequences given in **Supplementary Table 3**). The *V. montagnei* EF1α gene was used as the reference. The PCR cycles consisted of an initial denaturation (95°C/2 min) followed by 40 cycles of 95°C/15 s, 55°C/15 s, and 72°C/20 s. Relative expression levels were calculated using the 2^−△△CT^ method.

## Results

### The life cycle of the moss, *V. montagnei*, in culture

**Figure 1:**
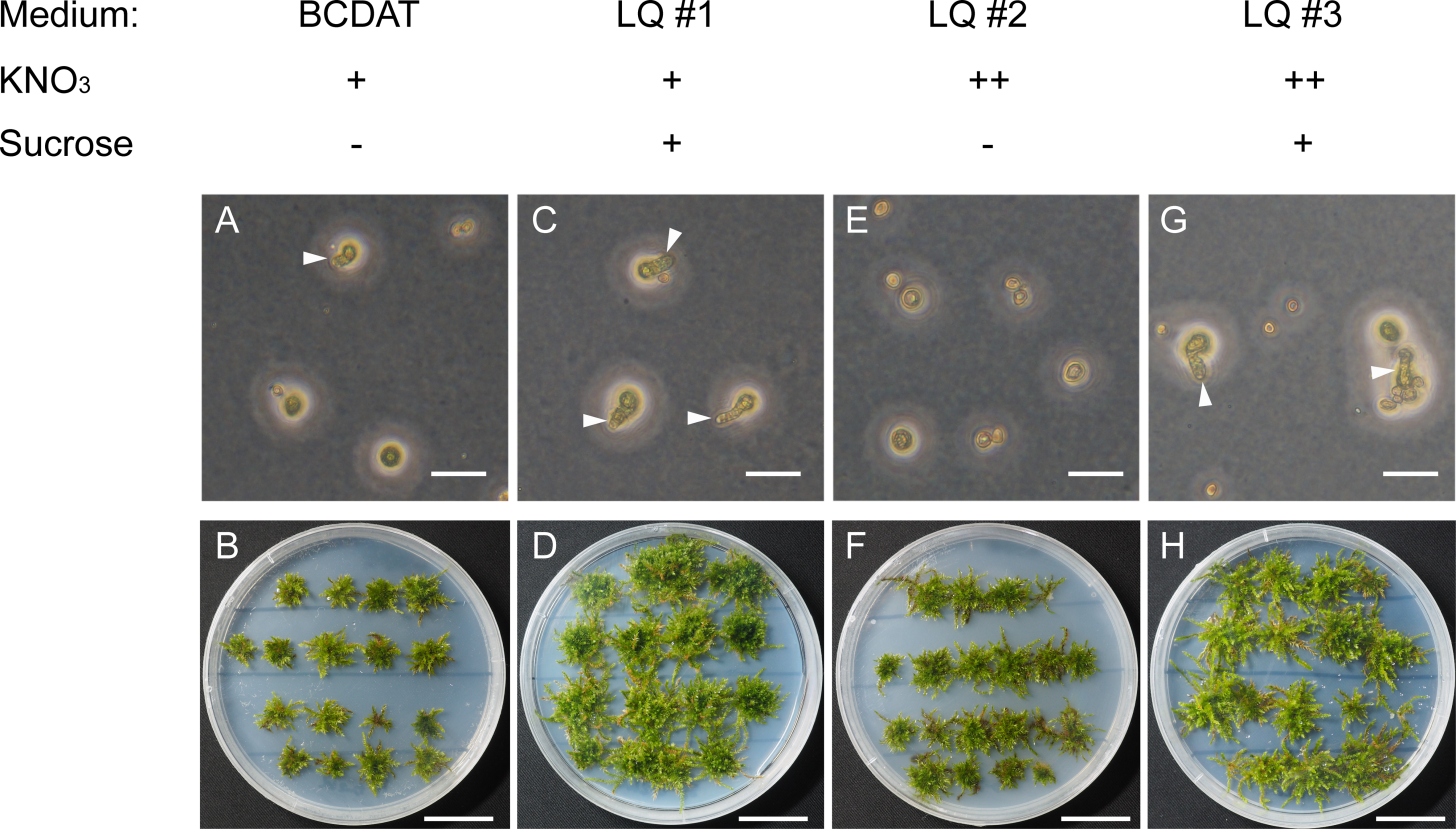
The germination of spores and growth state of *Vesicularia montagnei* (*V. montagnei*) on different medium. Spores germination **(A, C, E, G)** and gametophore plantlets development **(B, D, F, H)** on BCDAT medium with normal KNO_3_ concentration **(A, B)**, or with additional of sucrose (**C**, **D**, medium refer as LQ #1), or with elevation concentration of KNO_3_ [**(E, F)**, medium refer as LQ #2], or with additional of sucrose and elevation concentration of KNO_3_ [**(G, H)**, medium refer as LQ #3]. Bars in **(A, C, E** and **G)**, 50 μm; Bars in **(B, D, F** and **H)**, 20 mm.

*V. montagnei* is chosen in this study because of its ability to live in temperate, subtropical, and tropical Asia with different environmental conditions (Redfearn, 1990; Kürschner and Ochyra, 2014). These characteristics may make it suitable for broad application in various conditions. To establish a sustainable method of obtaining *V. montagnei* plants for greenery, we first tried to establish the axenic cultivation system. The original *V. montagnei* plants were cultivated in the greenhouse to develop sporophytes with capsules. Then, mature capsules were collected, and the containing spores were sterilized with 10% sodium hypochlorite, and germinated on standard BCDAT medium (Cove et al., 2009b). However, few spores could germinate (**Figure 1A**), and a poor growth state (**Figure 1B**) was observed. Next, we modified the BCDAT medium by adding sucrose (LQ #1), increasing the concentration of potassium nitrate (LQ #2) or both (LQ #3). The spores cultured on LQ #1 and LQ #3 showed a significant improvement in germination and growth of protonema and gametophyte compared to those cultured on standard BCDAT medium (**Figures 1C, D**). However, germination could hardly be observed on medium LQ #2 (**Figures 1E, F**), and there was no obvious difference in the growth of protonema and gametophyte on medium LQ #1 and #3 (**Figures 1A, B, G, H**). These results indicated that the addition of sucrose assisted the germination and growth of *V. montagnei*, but the increased concentration of potassium nitrate did not have a significant effect.

**Figure 2:**
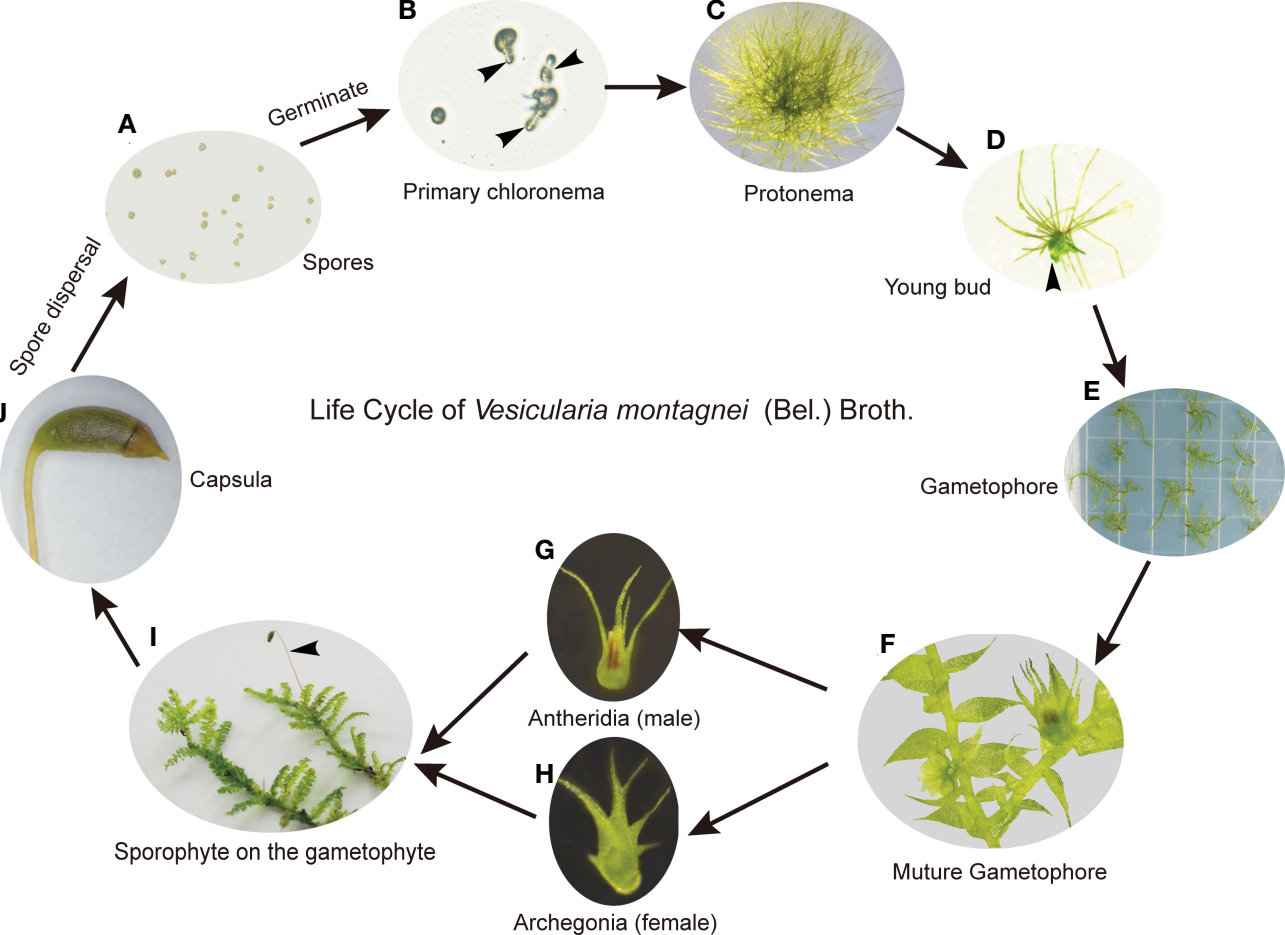
The life cycle of *V. montagnei* in culture. **(A)** Spores were sterilized and spread on the medium; **(B)** Spores start germinating, black arrows indicate the primary chloronema; **(C)** The development of protonema which including chloronema and caulonema cells; **(D)** The development of young bud from protonema, black arrows indicate the young bud; **(E)** The development of gametophyte; **(F–H)** Gametophyte become mature **(F)** and the development of antheridia **(G)** and archegonia **(H)**; **(I)** The development of sporophyte on the gametophyte, black arrows indicate the sporophyte; **(J)** Capsula develops on the sporophyte and become mature.

On the medium LQ #1, spores of *V. montagnei* (**Figure 2A**) began to germinate after 3 days of inoculation (**Figure 2B**) and formed filamentous chloronema and caulonema cells (**Figure 2C**). Buds that later developed to gametophores were formed after about 40 days of cultivation (**Figures 2D–F**). To complete the life cycle of *V. montagnei in vitro*, gametophytes were transferred to growth chambers with a temperature of 25°C and a short-day photoperiod (8h light/16h dark). Antheridia (**Figure 2G**) and archegonia (**Figure 2H**) started to emerge on the mature gametophytes at the axil on the stem of the same plant after about 50 days of culturing. Finally, the fertilized egg cell within the archegonia developed into a new capsule (**Figures 2I, J**). Thus, an axenic culturing system was successfully established, enabling the completion of the sexual life cycle of the moss *V. montagnei* in culture.

### Diploidization of genome of the moss *V. montagnei* increased the density and length of the rhizoids

**Figure 3:**
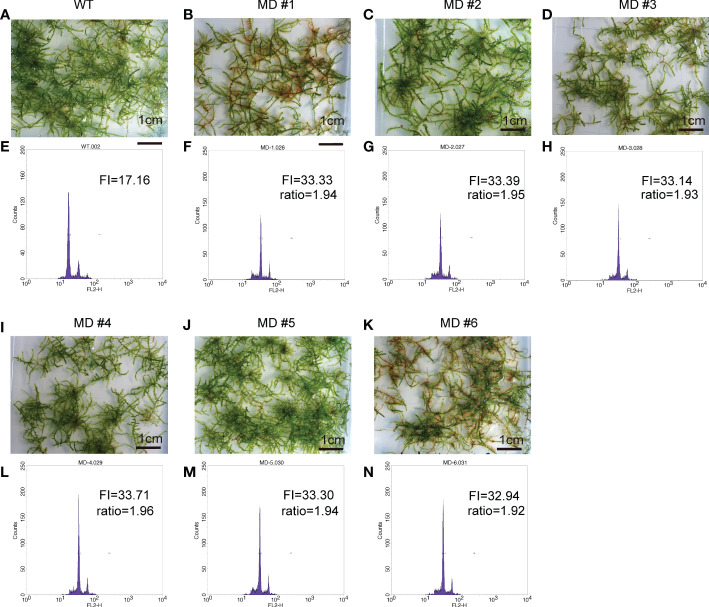
The overall view of the wild type (WT) *V. montagnei* and the diploid lines and the ploidy analysis. The phenotype of 3-month-old gametophyte growing on LQ #1 medium of WT **(A)** and diploid lines **(B–D**, **I–K)**; The ploidy analysis with flow cytometry of the WT **(E)** and diploid lines **(F–H**, **L–N)**, The x-axis reflects the relative fluorescence intensity of the stained nuclei.

To improve the application of *V. montagnei* in the greenery establishment, we attempted to induce diploid *V. montagnei* plants using a 0.3% (w/v) colchicine solution. After subculturing for 60 days, young protonema was immersed in colchicine solution for one week. A total of 153 treated lines survived the treatment were subjected to ploidy analysis with flow cytometry (FCM). Compared with that of the wild-type *V. montagnei* (WT, **Figures 3A, E**), 6 lines (**Figures 3B–D, I–K**) with cells containing doubled DNA content (MD, **Figures 3F–H, L–N**) and 3 lines with cells displaying semi-doubled DNA content (MSD, **Supplementary Figure 1**) were obtained in the initial screening. Subculture of the diploid lines was further analyzed with FCM, which validated the stability of the diploidy.

**Figure 4:**
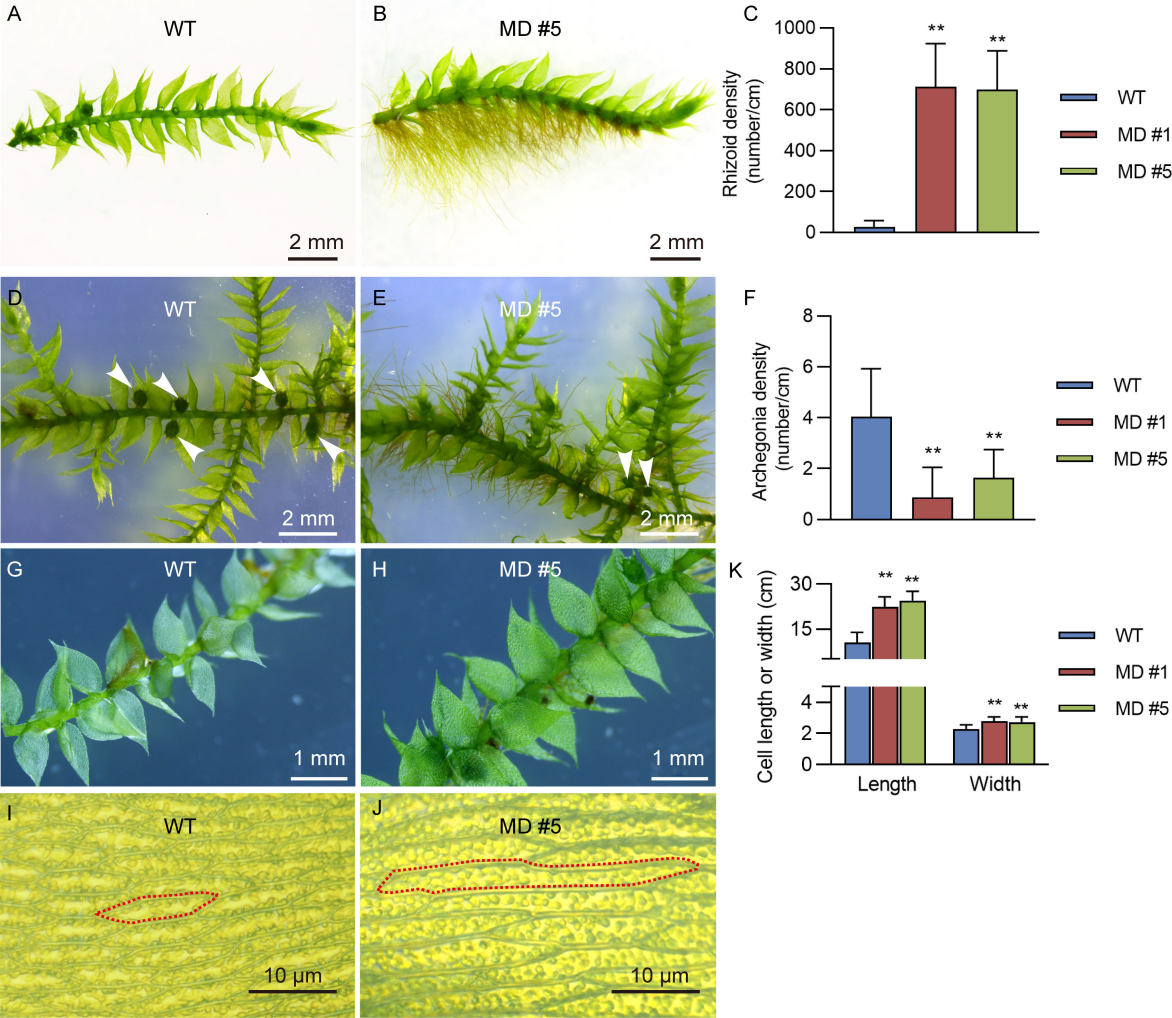
The enhancement of rhizoids growth and increasement of cell size of phylloid by diploidization. The comparison of growth of rhizoids **(A, B)**, archegonia **(D, E)**, leaf like phylloid **(G, H)** and cell size of phylloid **(I, J)** between 50-d-old WT and diploid line MD #5. The detailed data were showed in **(C, F, K)**; **p < 0.01, *t*-test; n = 20.

Interestingly, compared to the WT line, diploid *V. montagnei* lines displayed longer rhizoids with about 700 times higher density (**Figures 4A–C**). Moreover, diploidization significantly reduced the density of archegonium on the gametophyte (**Figures 4D–F**). In addition, slight difference in the phyloid size was observed (**Figures 4G, H**). Further investigation of the size of cells in the phyloid revealed that diploid lines had longer and wider cells (**Figures 4K, J**).

### Transcriptome changes of diploid line

To dissect the underlying mechanism behind the phenotypic changes following the diploidization, we conducted a transcriptome comparison between the WT and diploid lines. The whole gametophytes of the WT and diploid lines and rhizoids collected from the diploid line were subjected to RNA-sequencing using the Illumina platform. A total of 31.07 Gb of raw reads were ultimately obtained. Quality control showed that all of the samples showed Q30 levels higher than 95% (**Supplementary Table 1**). The transcriptome was *de novo* assembled, resulting in 41,520 unigenes and 75,694 transcripts.

**Figure 5:**
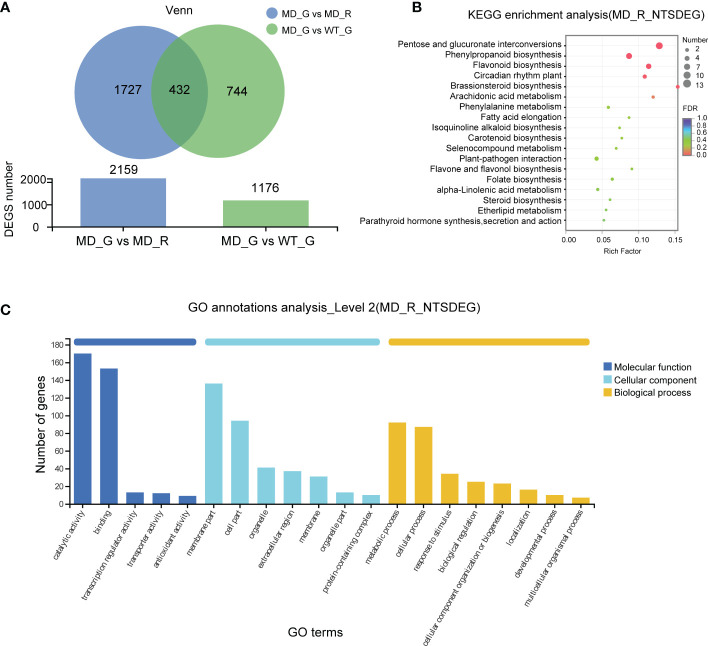
The transcriptome analysis of the WT and diploid line. **(A)** The numbers of differential expressed genes (DEGs) between different samples. The KEGG analysis **(B)** and GO annotations **(C)** of genes shared by MD-G vs MD_R and MD_G vs WT_G.

To investigate the transcriptional evidence for phenotypic changes in the diploid lines, we analyzed and identified 1176 differential expressed genes (DEGs) in gametophores between WT and diploid line (WT VS MD) (**Figure 5A**). In order to narrow down the range of putative target genes responsible for longer and denser rhizoid phenotype, we compared gene expressions between aboveground and below-ground parts of the diploid gametophyte (MD_G VS MD_R), resulting in 2159 DEGs (**Figure 5A**). The intersection of these two sets of DEGs consisted of 432 genes, which represent the putative candidate genes associated with the rhizoid phenotype changes in the diploid lines (**Figure 5A**). Furthermore, the Kyoto Encyclopedia of Genes (KEGG) enrichment analysis showed that the 432 DEGs were significantly enriched in the pentose and glucuronate interconversions, phenylpropanoid biosynthesis and circadian rhythm plant pathways (**Figure 5B**). According to the Gene Ontology (GO) analysis, the molecular function, cellular component, and biological process were the most enriched pathways (**Figure 5C**).

### Differentially expressed genes in the jasmonic acid (JA) biosynthesis pathway between WT and diploid *V. montagnei* lines

**Figure 6:**
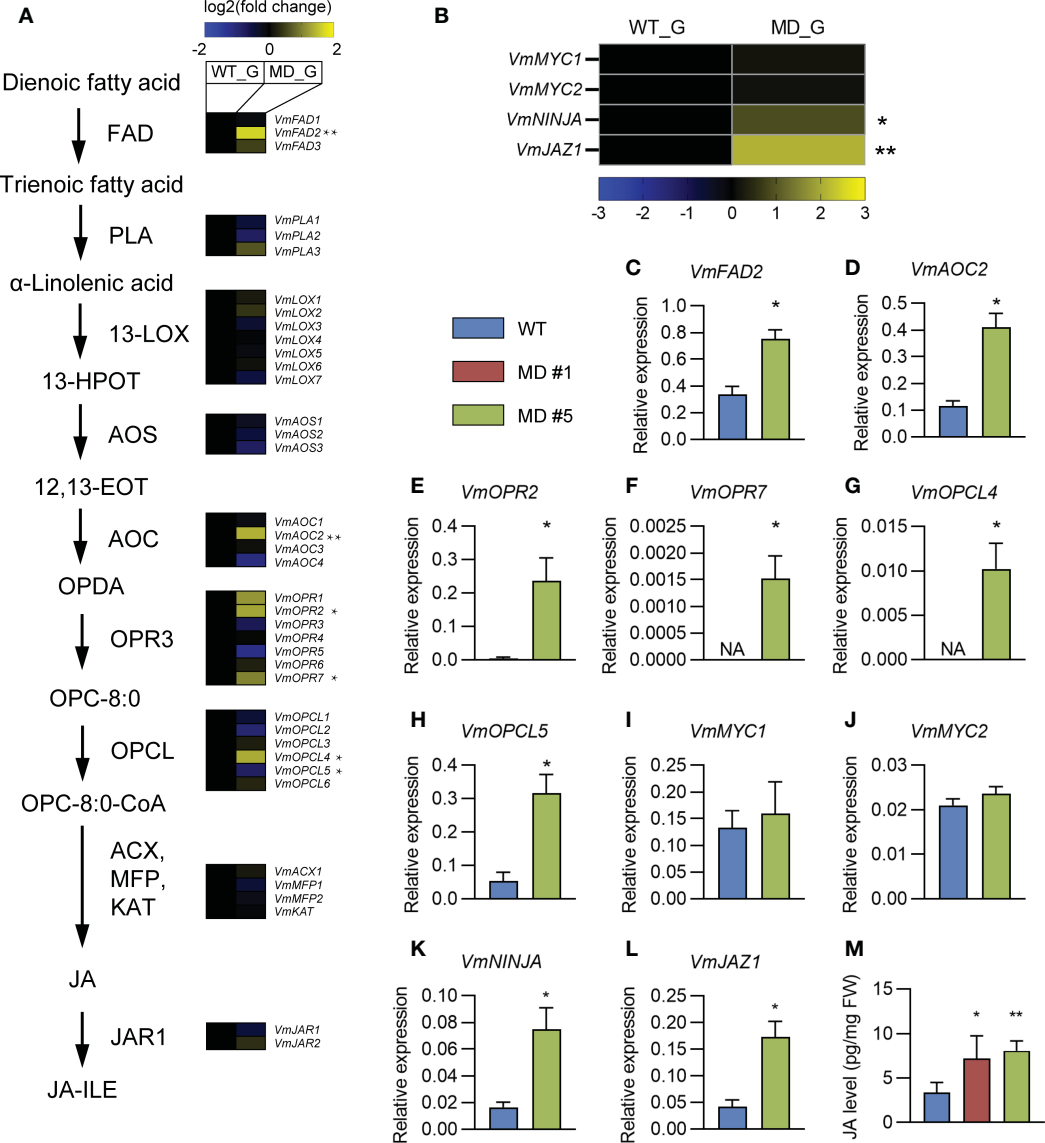
The comparison of JA biosynthesis and signaling pathways between WT and diploid line(s). **(A, B)** Comparison of transcription levels of genes in the JA biosynthesis **(A)** and signaling **(B)** pathways. **(C–L)** Comparing of the expression levels of indicated genes in WT and diploid plants. Whole plants of three-month-old plants was collected and used for quantitative real-time PCR (qRT-PCR). Data was shown as mean ± SD of three replicates. An actin gene was used for normalization. **(M)** Comparison of JA level in the gametophyte in the WT and diploid lines MD #1 and #5. **p < 0.01, *p < 0.05, *t*-test, n = 3. NA, not applicable because the expression levels of indicated genes are below the detection limit.

The alpha-linolenic acid metabolism is known as a precursor of JA biosynthesis (Pyo et al., 2022). The phenylpropanoid biosynthesis pathway is regulated by the plant hormone JA (Dong and Lin, 2021). The significant enrichments of DEGs in both pathways (**Figure 5B**) motivate us to pay a close attention on the effects of diploidization on the JA biosynthesis and signaling. BLAST analysis against the transcriptome of the *V. montagnei* was conducted to obtain the homologs in the biosynthesis pathway (*FAD*, *PLA1*, *LOX3*, *AOS*, *AOC3*, *OPR2*, *OPCL1*, *ACX5*, *MFP2*, *KAT2*, and *JAR1*) and the signaling pathway (*MYC2*, *NINJA*, and *JAZ1*) (**Supplementary Table 2**). Expressional analysis of these genes through RNA-seq showed that several homologs, like *VmFAD2*, *VmAOC2*, *VmOPR2*, *VmOPR7*, and *VmOPCL4* in the biosynthesis pathway were significantly enhanced in the diploid line compared with the WT, although higher expression of *VmOPCL5* was detected in the WT (**Figure 6A**). Moreover, two signaling genes, *VmNINJA* and *VmJAZ1* also showed higher expression levels in the diploid line (**Figure 6B**). These differentially expressed genes were validated with quantitative-PCR in the three-month-old plants (**Figures 6C–L**). Consistently, the JA level in the diploid line is significantly higher than that in the WT (**Figure 6M**). These results demonstrated that the diploidization increased the level of JA in plants, which is probably caused by the enhancement of the JA biosynthesis pathway.

## Discussion

Mosses are increasingly popular in establishing natural living landscapes in urban buildings or indoor environments. The stable and sustainable way of obtaining mosses for these applications demands a standardized *in vitro* cultivation system. The previous study reported that the development of cultivation systems for different peat moss species is also important in mitigating climate change (Zhao et al., 2019; Heck et al., 2021). In our study, the moss *V. montagnei* that can adapt to different climate environments was chosen to establish an *in vitro* cultivation system.

Our results showed that adding sucrose to the BCDAT medium promotes both germination and the growth state of *V. montagnei*, which differs from *P. patens* that grows well without external carbon source (Cove et al., 2009b). It was also reported that external sugars had a negative impact on protonemal development and shoot multiplication of the moss *Atrichum undulatum* (Sabovljevic et al., 2005). Thus, different mosses may respond differently to exogenous sugars. However, in different species from the same genus of *Sphagnum*, sucrose- or glucose-addition has been consistently found to boost growth (Simola, 1969; Graham et al., 2010; Beike et al., 2015). Despite carbon source, nitrogen source has also been reported to be critical for determining growth in diverse bryophytes (Basile, 1975). In our study, simply increasing the concentration of KNO_3_ did not enhance the growth of *V*. *montagnei* (**Figure 1B-H**). An appropriate ratio between sucrose and ammonium nitrate is critical for the growth of protonema of the moss *Polytrichum commune* (Yu et al., 2008). The addition of sucrose and ammonium was able to increase the biomass of *Sphagnum palustre* (Beike et al., 2015). Moreover, different nitrogen sources also affected the growth rates, bud production rates, and plant morphology in the cultivation of *Splachnum ampullaceum* (Gonzalez et al., 2006). Our optimized medium may provide valuable information for the studying other *Vesicularia* species. In addition to sucrose and nitrogen sources, plant hormones were used to adjust the growth of mosses. The combinations of BA and IBA with different concentrations were used to obtain of moss *Bruchia vogesiaca* in different developmental phases (Sabovljević et al., 2012b). It will be interesting to investigate the effect of plant hormones on the growth of *V. montagnei* in the future.

Various media types, such as BCD, Murashige and Skoog (MS), and Knop, have been used for growing different species of mosses (Reski and Abel, 1985; Cove et al., 2009b; Sabovljević et al., 2012a; Pandey et al., 2014). Previous studies compared the effectiveness of BCD and half-strength MS (MS/2) media in cultivating *Bruchia vogesiaca* (Bruchiaceae) and *Entosthodon hungaricus* (Funariaceae) (Sabovljević et al., 2012a; Sabovljević et al., 2012b). It was found that MS/2 medium was the most suitable for the propagation and subculturing of *E. hungaricus* (Sabovljević et al., 2012a). However, *B. vogesiaca* had better growth in terms of protonema diameter and bud initiation when cultivated in the BCD medium (Sabovljević et al., 2012b). In this study, the successful axenic cultivation of *V*. *montagnei* for its entire life cycle was achieved on the BCDAT solid medium with added sucrose (**Figure 2**). It appears that different species of moss may have varying preferences for the types of media. Our development of this axenic cultivation system has enabled the propagation and multiplication of homogeneous and insect-free *V*. *montagnei* plant materials. And most importantly, this system allows for the establishment of several lines of diploid *V*. *montagnei* plants with an improved rhizoid system (**Figures 4A–C**). These lines may hold promising potential for greenery applications. It has been reported that cultivation of *Sphagnum* mosses in the liquid Knop medium yielded high amounts of biomass (Beike et al., 2015; Zhao et al., 2019; Heck et al., 2021). Conducting further investigations to test or modify the liquid medium for the purpose of increasing the biomass of *V. montagnei* moss can be helpful for its application in greenery establishment.

Artificial-induced polyploidization is a commonly used technique in horticultural plant breeding, which usually produces larger organs such as flowers, fruits, seeds, leaves, stems, and roots in vascular plants (Eng and Ho, 2019). This phenomenon is termed as “giga” effect. Our study found that the diploidization of the early landed moss *V. montagnei* also resulted in enhanced growth of rhizoids (**Figures 4A, B**) and slightly larger leaf-like phyloid (**Figures 4G, H, I, J**). These results provided an additional example of the “giga” effects of polyploidization in early landed plants. However, a previous study in *P. patens* showed that diploidization significantly decreased plant growth rate and dry weight (Gabriele et al., 2005). These different phenotype changes indicated the divergence of the genome structure between different moss species. Our results indicated that artificial-induced polyploidization has great potential for developing diploid moss lines with improved growth performances.

In this study, JA was detected in the fresh samples of both the WT and diploid lines of *V. montagnei* (**Figure 6H**). Consistently, several putative homologs of the JA synthesis pathway were detected in the transcriptomic analysis (**Figure 6A**), which may form a complete synthesis pathway in the *V. montagnei*. Similarly, previous phytohormone profiling of several bryophytes (including 6 and 24 species of liverworts and mosses, respectively) successfully detected JA with the concentration range from 1.20 pmol/g FW (*Sphagnum* sp.) to 78.80 pmol/g FW (*Pogonatum urnigerum*) (Záveská Drábková et al., 2015). In our study, JA-Ile is not detectable in *V. montagnei*, which may be caused by the extremely low level or the inactive JAR1-like enzyme. The former reason is more likely because JA-Ile is far less abundant than JA and occurs at very low amounts (almost close to the limit of detection) in several moss species (*Pellia endiviifolia*, *Sphagnum compactum*, *Sphagnum* sp., and *Polytrichum commune*) (Záveská Drábková et al., 2015). However, studies in the *P. patents* and the liverwort *Marchantia polymorpha* could only detect the precursor OPDA but not JA or the conjugate JA-Ile (Stumpe et al., 2010; Ponce De León et al., 2012; Yamamoto et al., 2015), even though the *P. patens* genome contains several OPR-like genes (Breithaupt et al., 2009; Li et al., 2009). These results suggested that there may be evolutionary divergences in the JA biosynthesis pathway among the bryophytes. Further investigation of the enzymes responsible for the metabolism downstream of OPDA (such as OPR-like and OPCL) may help to clarify this.

The JA-Ile is the bio-active form of JA in the vascular plants and is able to induce the expression of several JA signaling genes through the negative feedback loop (Chico et al., 2008). Interestingly, JA-Ile was not detected in the moss *V. montagnei*, but the signaling homologs *VmJAZ1* and *VmNINJA* showed increased expression levels in the diploid lines with elevated JA content (**Figures 6K, M**). This suggests that the bio-active jasmonate in the moss *V. montagnei* is likely not JA-Ile. Notably, a study in *Nicotiana attenuata* found that JA-Ile is not the only active oxylipin signal molecule (Wang et al., 2008). While the JA-Ile application fully restored the resistance to *Manduca sexta* feeding in *JAR4*/*6*-silenced plants (impaired in JA-Ile synthesis), it only partially restored resistance in *LOX3*-silenced plants (deficient in JA–Ile and other oxylipins). Further search for the bio-active jasmonate besides JA-Ile will be very interesting. Our investigation provided evolutionary evidence for the existence of other bio-active jasmonate besides JA-Ile and improved our understanding of jasmonate biosynthesis.

It was proposed that rhizoid in moss gametophytes and root hair on the roots of vascular plant sporophytes were close related in the evolutionary perspective (Jones and Dolan, 2012). Because a similar gene regulatory network controls the development of rhizoids and root hairs. In the vascular plant Arabidopsis, JA has been shown to have a pronounced effect on promoting root hair formation (Zhu et al., 2006; Han et al., 2020). Consistently, our study found a significant increased rhizoid density associated with the accumulation of JA content in the diploid lines of *V. montagnei* (**Figures 4A–C**). These results suggested that JA has a conserved function in regulating the development of both rhizoids and root hairs. Based on our findings, it is promising to enhance the rhizoid growth through the direct application of JA *in vitro*, which may improve the ability of the plants to adhere to the surfaces for greenery decoration purposes.

## Data availability statement

The datasets presented in this study can be found in online repositories. The names of the repository/repositories and accession number(s) can be found below: https://ngdc.cncb.ac.cn/gsa, GSA: CRA009372.

## Author contributions

LL designed and supervised the study. QL and YH performed most of the experiments and analyzed the data. ZC contributed in the diploid induction. ZX analyzed the RNA-seq data. ZC, HL, and CW contributed in the preparing of mosses. YH and QL drafted the manuscript. LL, LD, and HY revised the manuscript. All authors contributed to the article and approved the submitted version.
