# Supplementary material for: Axenic in vitro cultivation and genome diploidization of the moss Vesicularia montagnei for horticulture utilization
Source: Front Plant Sci. 2023 Mar 20;14:1137214. doi: 10.3389/fpls.2023.1137214 (PMC10067734; doi:10.3389/fpls.2023.1137214)
Supplement: Supplementary file 1 [file DataSheet_1.pdf]

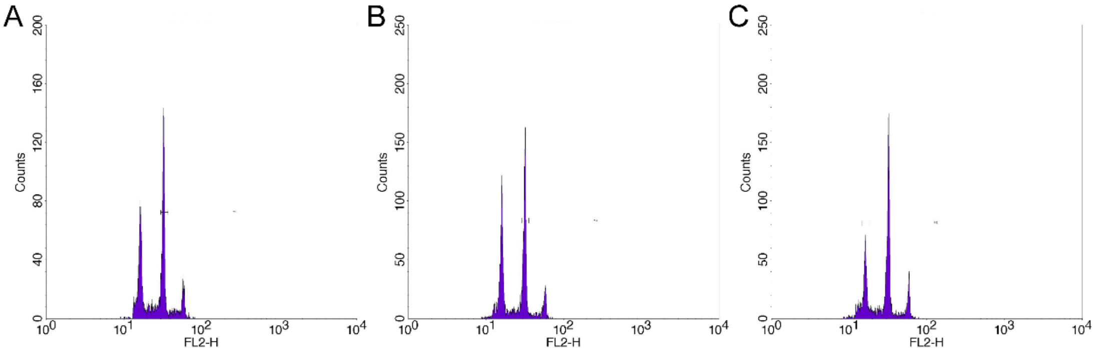

Supplementary Figure 1. Three lines of colchicine solution treated *V. montagnei* showed semi-doubled genome revealed by flow cytometry.

The ploidy analysis with flow cytometry of three independent semi-doubled lines **(A-C)**, The x-axis reflects the relative fluorescence intensity of the stained nuclei.
